# Tunable Structure and Properties of Co-Evaporated Co–C60 Nanocomposite Films

**DOI:** 10.3390/nano15100715

**Published:** 2025-05-09

**Authors:** Ziyang Gu, Yiting Gao, Zhou Li, Weihang Zou, Keming Li, Huan Xu, Zhu Xiao, Mei Fang

**Affiliations:** 1 School of Materials Science and Engineering, Central South University, Changsha 410083, China; 223111008@csu.edu.cn (Z.G.); 2401110173@stu.pku.edu.cn (Y.G.); lizhou6931@163.com (Z.L.); xiaozhumse@163.com (Z.X.); 2 Hunan Key Laboratory for Super-microstructure and Ultrafast Process, School of Physics, Central South University, Changsha 410083, China; 8201220418@csu.edn.cn (W.Z.); 242231002@csu.edu.cn (K.L.); 242212080@csu.edu.cn (H.X.); 3 State Key Laboratory for Powder Metallurgy, Central South University, Changsha 410083, China; 4 Key Laboratory of Non-ferrous Metal Materials Science and Engineering, Ministry of Education, Changsha 410083, China

**Keywords:** magnetic nanoparticle, co-evaporation, composite film, magnetoresistance

## Abstract

Magnetic nanoparticles (NPs) hold great promise for both fundamental research and future applications due to their unique structural features, high specific surface area, and tailored physical properties. Here, we present a convenient thermal co-evaporation approach to deposit Co–C60 composite films with controlled composition, structure, morphology, and tunable performances, specifically designed for spintronic device applications. By tuning the growth rates of Co and C60 during co-evaporation, the composition of the films can be tuned with different ratios. With a Co/C60 ratio of 5:1, ~300 nm clusters are formed in the films with increased coercivity compared with pure Co films, which is attributed to the interfaces in the composite film. The magnetoresistance (MR), however, becomes dominated by organic semiconductor C60 with ordinary magnetoresistance (OMAR). By increasing the composition of C60 to the ratio of 5:2, the particle diameter decreases while the height increases dramatically, forming magnetic electrodes and, thus, nano-organic spin valves (OSV) in the composite films with giant magnetoresistance (GMR). The work demonstrates a versatile approach to tailoring the structural and functional properties of magnetic NP-composite films for advanced spintronic applications.

## 1. Introduction

Magnetic nanoparticles (NPs) are receiving increasing attention due to their unique magnetic, electronic, optical, and chemical properties, making them suitable for a wide range of applications such as memory and spintronic devices, electrocatalysis, and so on [1,2,3,4]. Cobalt nanoparticles are particularly popular because of the high saturation magnetization and susceptibility, as well as the long spin relaxation time (150 ns [5]) of ferromagnetic metal Co, all of which are desirable for spin-polarized charge transport in spintronic applications. It was reported that by depositing a cobalt NP layer between the organic and the ferromagnetic (FM) layers in organic spin valves (OSVs), the magnetoresistance (MR) can be improved dramatically because of the impeding interdiffusion at the interface [6,7]. Additionally, the magnetocrystalline anisotropy of cobalt was found to be modulated by organic materials such as benzene, cyclooctatetraene, and naphthalene, which was attributed to the interface hybridization. [5,8] The out-of-plane magnetic anisotropy was reported to be greatly enhanced by hybridizing the d_z_^2^ orbitals of Co on the surface with the p_z_ orbitals of Carbon. [8] Recently, the spinterface science has been proposed: The discrete energy level of organic molecular can be broaden or shifted because of the interface hybridization [9,10]. Even at the FM/Inorganic interface, spin-dependent band-splitting would reverse the spin polarization in ferroelectric tunnel junctions [11,12]. Developing interface-dominated spintronic devices becomes particularly important. Nanoparticles embedded within organics can maximally increase the interface effect and, therefore, predominate the properties of the spintronic devices with additional functions and novel properties.

Significant progress has been made in developing methods for preparing cobalt nanoparticles, including cost-effective and scalable chemical approaches like chemical reduction of oxides [13,14,15], spray pyrolysis [16,17], sol-gel methods [18,19], and decomposition of cobalt salts [20], etc. Additionally, vapor depositions have also been developed because of the advantages in high quality and film-forming ability, where the particle diameter can be adjusted by the related factors during deposition. For instance, using helium as a carrier gas [21], 10–25 nm spherical and rod-shaped cobalt and cobalt oxide hybrid nanoparticles with high saturation magnetization (95 Am^2^/kg) and large coercivity (822 Oe) were obtained by chemical vapor deposition. By controlling the current and the pressure of argon gas in arc plasma evaporation [22], pure Co nanoparticles were fabricated by melting the Co metal block in a vacuum with particle sizes modulated within a range of 28–70 nm.

Herein, we propose a co-evaporation method for preparing Co nanoparticle–organic composite films for practical spintronic device applications. By co-evaporating Co and C60 at varying growth rates, the film compositions are controlled, resulting in tunable morphology and electric structures. These changes further induce modifications in magnetic and electric properties, driven by the formation of Co nanoparticles. The work provides a convenient and controllable approach to growing Co nanoparticles in composite films with tunable particle size and magnetic properties, demonstrating their potential for spintronic applications.

## 2. Materials and Methods

Pure cobalt thin films (Sample Co) and the composite films were deposited on alumina (0001) substrates using thermal evaporation in a high-vacuum system with a base pressure of ~2 × 10^−6^ Pa. The growth rate of Co was 0.3 Å/min monitored by a SCIENS SI-TM608 quartz crystal oscillator (Shenyang, Liaoning, China), which was calibrated by the final film thickness measured with a Bruker DektaXT profilometer (Tucson, AZ, USA). The Co–C60 composite thin films were prepared via co-evaporation, in which the growth rate of Co remained 0.3 Å/min, while the growth rate of C60 was tuned to obtain different compositions of the films. The composite films with ratios of Co-to-C60 at 5:1 (Sample D1) and 5:2 (Sample D2) were prepared with C60 growth rates of 0.06 Å/min and 0.12 Å/min, respectively. The pure C60 film (Sample C60) was deposited with a growth rate of 3 Å/min.

Raman spectra were obtained using a Thermo Scientific DXR3 Raman microscope (Madison, WI, USA) with a 532 nm laser excitation. A PHI VersaProbe 4 (Chanhassen, MN, USA) X-ray photoelectron spectroscopy (XPS) with Al Kα radiation was employed to detect the oxidation states of Co in composite films. The spectra were calibrated using the C1s peak arising from adventitious carbon with a binding energy of 284.6 eV. The surface morphologies of the films were characterized by a BENYUAN CSPM5500 (Shenzhen, Guangdong, China) atomic force microscope (AFM) using taping mode. The crystal structures of the composite films were studied with a JEOL JEM-F200 (Tokyo, Japan) transmission electron microscope (TEM). The magnetic hysteresis loops of the films were detected by a custom-built magneto-optical Kerr effect (MOKE) setup with a laser wavelength of 670 nm. The transport properties of the films were measured by a Keithley 2400 source meter, with a controlled temperature and magnetic field provided by a Quantum Design physical properties measurement system (PPMS, San Diego, California, USA). The 20 nm thick Co–C60 composite films were patterned into 8 × 1 mm^2^ strips using a shadow mask for electric transport measurements.

## 3. Results and Discussion

### 3.1. Electronic Structure of the Films

**Figure 1:**
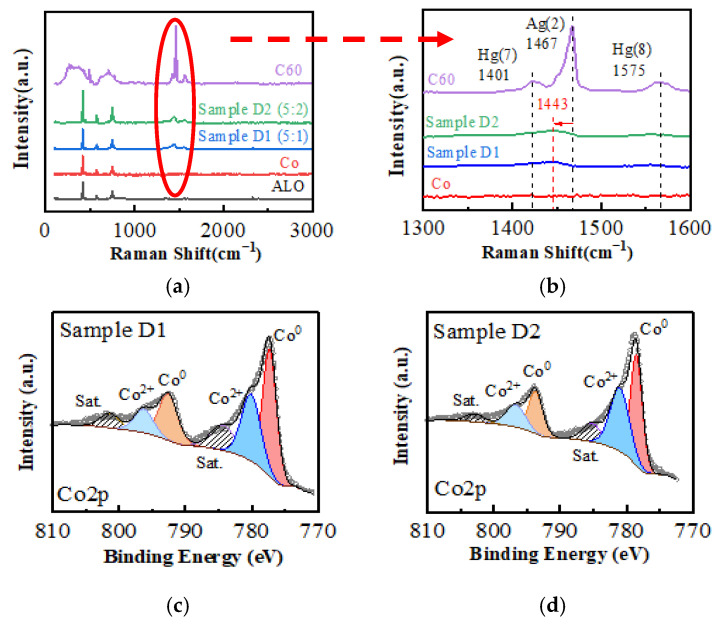
Electronic structure investigations of the films. (**a**) Raman spectra of the composite films compared with the alumina (ALO) substrate, pure C60 and pure Co films, and (**b**) the characteristic peaks of C60. The shifts of C60 Ag (2) peaks can be observed due to the electron transfer at the Co–C60 interface. (**c**) and (**d**) show the XPS analysis for the Co2p peaks of Samples D1 and D2, respectively.

To address the possible interface hybridization, the electric structures of the composite films were investigated, as shown in the Raman spectra in Figure 1a,b. Three characteristic peaks of C60 were found: Ag (2) at 1467 cm^−1^, Hg (7) at 1401 cm^−1^, and Hg (8) at 1575 cm^−1^, corresponding to the tangential vibrations of the carbon atoms in the hexagonal ring (Ag (2)) and in the pentagonal ring (Hg peaks) of C60, respectively. The Ag (2) peak, which stands for the molecular valence of C60 [23], shifts to a shorter wave number of 1443 cm^−1^ in the composite films. This noticeable red shift can be attributed to the electron transfer at the Co/C60 interface [24,25]. Since the Fermi energy of electrons in Co is higher than the high occupied molecular orbit (HOMO) energy level of C60 [26], electrons can be transferred from Co to the antibonding orbitals of C60 at the interface. This would lower the bonding energy level of C60 and lead to the observed red shift of the Ag (2) peaks. It has been reported [25,27] that in the metal–C60 polymer system, the transfer of one electron from metal atoms to a C60 molecule would decrease the wavenumber of C60 Ag (2) peak by 6 cm^−1^. In the present work, the observed displacement of 24 cm^−1^ (from 1467 cm^−1^ to 1443 cm^−1^) indicates that there might be an average of 4 electrons transferred from Co to a single C60 cage.

To confirm the electron transfer in the Co–C60 composite films, the oxidation states of Co were further investigated via XPS, as shown by the Co2p peaks in Figure 1c,d. After peak fitting, both samples exhibit Co^0^ peaks (located at 771.0 and 792.7 eV) and Co^2+^ peaks (located at 781.0 and 796.5 eV), respectively. However, the peak area ratios are different: in Sample D1 (5:1), the total peak area ratio of Co^2+^ to Co^0^ is 0.4372, whereas in Sample D2 (5:2), this ratio increases to 0.8547. The finding suggests that the amount of oxidized Co in Sample D2 is approximately double that in Sample D1. This observation aligns well with the doubled C60 doping concentration in Sample D2. As deduced from the Raman spectra, the increase in the number of C60 cages would enable more electrons transferred from Co to C60. Consequently, the amount of oxidized Co increases.

### 3.2. Microstructure of the Films

**Figure 2:**
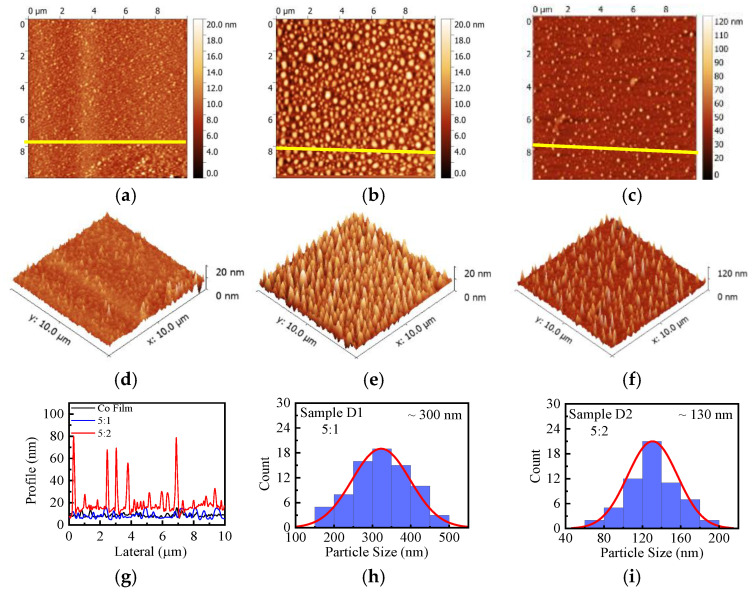
Surface morphology of the films: (**a**–**c**) 2D and (**d**,**e**) 3D AFM images for pure Co films (**a**,**d**), Sample D1 (**b**,**e**), and Sample D2 (**c**,**f**). (**g**) Line profile analysis of films at measurement positions marked by yellow lines in their corresponding 2D images (**a**–**c**). Size distributions of Co NPs in the composite films of Samples D1 (**h**) and D2 (**i**), respectively.

The doping of C60 could dramatically change the morphology of the films, as shown by the AFM images in Figure 2 and in the information listed in Table 1 for comparison. The pure cobalt film (Figure 2a,d) has a relatively flat surface with a root-mean-square (RMS) roughness of approximately 1 nm and few clusters. After doping with 16 vol.% C60 (Sample D1), a large number of clusters were observed with a diameter of ~300 nm, and the surface roughness increased, as shown by the 2D and 3D images in Figure 2b,e. By further increasing the doping concentration of C60 to 30 vol.%, Sample D2 (Figure 2c,f) shows that the cluster diameter decreases to ~130 nm, while the height continues to increase. Line profile analyses of the three samples are shown in Figure 2g, with the measurement positions marked by yellow lines in their respective 2D images (Figure 2a–c). Clearly, the height of the clusters in Sample D2 increases significantly to approximately 70 nm, which is ~10 times greater than that of the other films.

**Figure 3:**
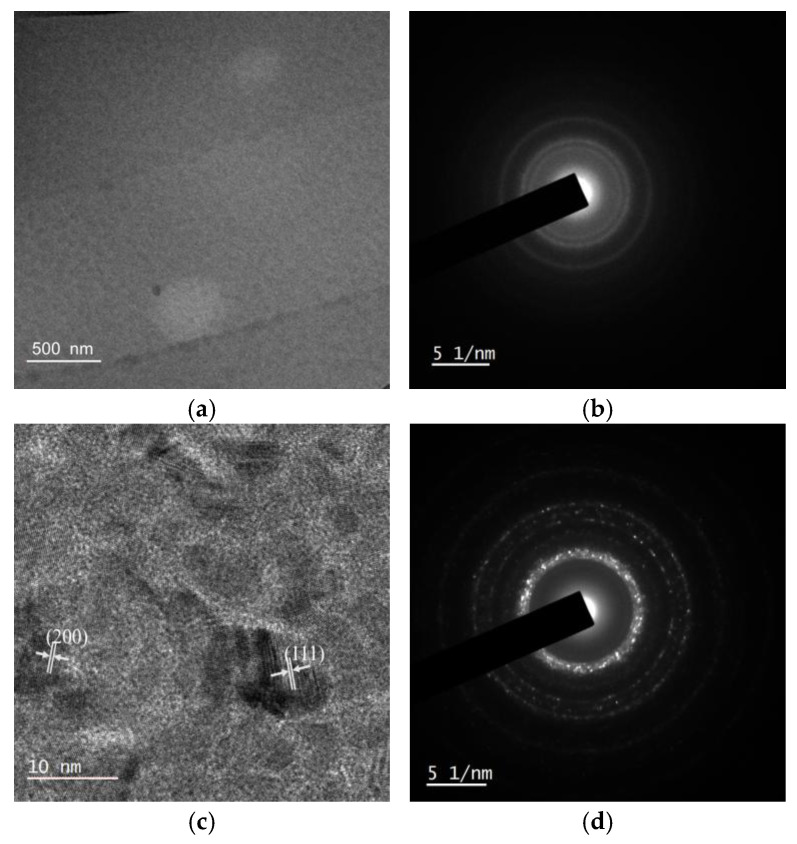
TEM images of the composite films: (**a**) Sample D1 and (**b**) the corresponding SAED pattern, and (**c**) Sample D2 and (**d**) the corresponding SAED pattern.

The crystallinity of the composite films was further investigated via TEM, the results of which are shown in Figure 3. For Sample D1, it is challenging to identify C60 from Co, and amorphous diffraction rings are observed in selected area electron diffraction (SAED). From the high-resolution TEM images of Sample D2, ~10 nm-sized crystals of Co can be clearly observed with high brightness boundaries. These high-brightness grain boundaries are considered region filled with lower conductive C60, indicating that the Co crystals are separated by C60. The improved crystallinity is further confirmed by the SAED patterns: The diffraction spots from crystals can be identified from the diffraction rings.

These observations suggest that C60 could dramatically change the crystallization process of cobalt during co-evaporation. According to the Volmer–Weber growth mechanism [28], Co atoms have a tendency to grow on the same material to lower the system energy and form clusters during the co-deposition process. Therefore, the Co clusters increase in both number and size for Sample D1 compared with the pure Co film. The further increase of the C60 deposition rate in Sample D2 would decrease the concentration of cobalt in the in-plane direction, leading to the out-of-plane growth of Co with a reduced diameter, but increased height and better crystallinity. It can be predicted that when the volume fraction of C60 exceeds a certain value, cobalt particles can be encapsulated by C60, resulting in nanoparticles embedded in films.

### 3.3. Magnetism of the Films

**Figure 4:**
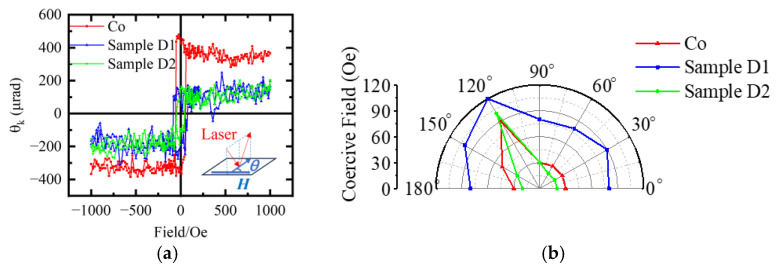
Magnetic properties of the pure cobalt film and the composite films: (**a**) Magnetic hysteresis loops from MOKE. The inset shows the MOKE measurements with the in-plane magnetic field. The θ is the angle between the field direction and the incident laser plane. (**b**) Angular dependent coercivity of the films.

The electric-structural (Figure 1) and microstructural (Figure 2 and Figure 3) changes in the films lead to significant modifications in their magnetic and electric properties. Figure 4 shows the in-plane magnetization hysteresis loops from MOKE for films with and without C60 doping. The rectangular hysteresis loops indicate that the easy magnetization axis is along the in-plane direction of the films. Since the Kerr rotation ($\theta_{K}$) depends on the measuring conditions, we detect the coercivity of the films from the MOKE loops and analyze its dependence on the in-plane magnetic field directions. As shown in Figure 4b, the coercivity and its angular dependence can be tuned by manipulating the concentration of C60. When *θ* = 0°, the magnetization of the films lies in the laser-plane of the incident and reflected lasers, corresponding to the longitudinal MOKE (L-MOKE). For the pure cobalt film, the coercivity is approximately 30 Oe. With 16 vol% C60 doping, the coercivity increases to around 80 Oe in Sample D1, but decreases to 20 Oe in Sample D2 with 30 vol% C60 doping. These changes in coercivity are attributed to the crystallinity of the films and its effects on the magnetization process. The Co films are poly crystalline structured with a crystalline size much smaller than the domain wall width δ_w_ = 24 nm [29]. The exchange coupling in the films across the grain boundaries and the coercivity correspond to the magnetization switching energy dominated by the movement of the domain wall at the grain boundaries. With C60 doping in Sample D1, the composite film becomes amorphous (Figure 3). C60 molecules could contribute to the pinning effect of magnetic domain motion, leading to the increased coercivity. The amorphous structure would decrease the anisotropy of the films, as well, leading to the reduced variation in its coercivities measured at different *θ* values. However, in Sample D2, Co nano crystals are formed with better crystallinity and surrounded by C60. The magnetization process would be dominated by the domain wall motion in nanocrystals similar to that in Co films.

### 3.4. Electric and Magnetic Transport Properties

**Figure 5:**
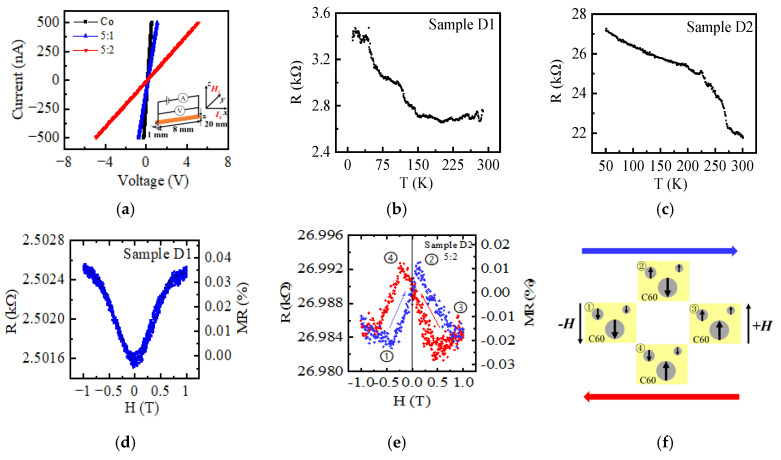
Electrical and magnetic transport properties of the films. (**a**) IV curves of the films measured at 10 K, determined from strips with a length of 8 mm and width of 1 mm. The geometry of the films and the architecture of the measurements are shown in the insert figure. (**b**,**c**) The temperature-dependent resistances for Samples D1 and D2, measured with a constant current of 100 nA. (**d**,**e**) Magnetic field-dependent resistance for Samples D1 and D2, measured with a constant voltage of 1 V and 3 V, respectively. The arrows show the directions of magnetic field sweeping. (**f**) The two-spin-channel model for the nano OSVs in Sample D2. The blue and red arrows indicate the sweeping direction of magnetic field.

The transport properties of these films were further investigated for advanced spintronic applications, the results of which are shown in Figure 5. The I-V curves shown in Figure 5a are linear with slopes represent the film resistances, which increased from 757.6 Ω for pure Co films to 1824.8 Ω and 10,021.1 Ω for the doped films of Samples D1 and D2, respectively. The corresponding conductivity ($\sigma =\frac{L}{RS}$) of the films can then be calculated based on the strip geometry of 8 × 1 mm^2^ and thickness of 20 nm (L=8 mm, S=1 mm×20 nm); the detailed results are listed in Table 1. The increase of the resistance (decreased conductivity) by C60 doping is attributed to the high resistivity of semiconductive C60, as well as the increased electron scattering due to the formation of the Co–C60 interfaces (Figure 3). Additionally, the temperature-dependent resistance measurements reveal that the doped films exhibit semiconducting behavior: the resistance decreases as the temperature increases, as shown in Figure 5b,c. In metals, the conductivity relies on the motion of free electrons, and the resistance primarily stems from the lattice scattering of electrons. An increase in temperature would enhance the lattice vibrations and result in electron scattering with higher resistance [30]. In semiconductors, in contrast, the conductivity involves electron hopping, a process that can be enhanced by thermal energy [31]. The observed results indicate that the doped C60 plays an important role in the electrical transport process in the composite film.

Furthermore, considering the formation of magnetic Co nanoparticles in the composite films, the spin-dependent transport properties were investigated. The resistance and the magnetoresistance ($MR=\frac{R_{H}-R_{0}}{R_{0}}$) of the composite films were detected by sweeping the out-of-plane magnetic field, the results of which are shown in Figure 5d,e. Sample D1 exhibits positive MR without hysteresis, indicating that the organic magnetoresistance (OMAR) effect dominates the transport of electrons. This result is consistent with the magnetic field effects in polymer-fullerene blends [32], where the spin blocking under the external magnetic field would decrease the current according to the bipolaron mechanism [33,34]. The further increase of C60 concentration in Sample D2, however, shows significant changes in the magnetoresistance with an obvious hysteresis effect in the low magnetic field region, which is similar to the hysteresis curves reported in organic spin valves (OSVs) [35,36]. We attribute these phenomena to the spin-dependent transport in Sample D2, where the magnetic Co nanocrystals (Figure 3c,d) play roles as magnetic electrodes with different coercivities for parallel and antiparallel magnetization alignment during the sweeping of the magnetic field, and the C60 in between serves as the spacer in OSVs. According to the two-spin-channel model [37], the resistances are different when the magnetic moments of two cobalt particles are in parallel (R_P_, states ①③) or antiparallel (R_AP_, states ②④) alignment, as shown by the schematic structure in Figure 5f. The diameter and the orientation of cobalt nanocrystals in D2 affect the magnetization and the coercivity, leading to the formation of nano-sized spintronic devices in the films. In the higher magnetic field region, the magnetic moments of Co particles are aligned with the external magnetic field, and the resistance is again dominated by the OMAR of C60.

## 4. Conclusions

We developed a convenient thermal co-evaporation method for depositing magnetic–organic semiconductor nanocomposite films with a controlled fraction of components, morphology, structure, and tunable magnetic and electric properties. By doping C60, clusters form in the films with tunable diameters, and, therefore, the magnetic and electric properties become tunable. Specifically, the magnetoresistance of the Co–C60 composite films can be tuned from the OMAR of organics to spin valves for Samples D1 and D2, respectively. These results suggest that the co-evaporation process can be used to prepare stable magnetic composite films with tunable and spin-dependent transport properties, which have potential for future spintronic device applications.

## Figures and Tables

**Table 1:** Sample information.

Sample ID	Average Size (nm)	RMS Roughness (nm)	Peak-Valley Height (nm)	Coercivity 0° (Oe)	Coercivity 120° (Oe)	Conductivity 10 K (S/m)
Sample Co	--	1	4	30	90	5.3 × 10^5^
Sample D1	300	2	10	80	120	2.2 × 10^5^
Sample D2	130	12	72	20	100	4.0 × 10^4^

## Data Availability

The raw data supporting the conclusions of this article will be made available by the authors on request.

## Abbreviations

The following abbreviations are used in this manuscript.

θ_k_	Kerr rotation
δ_w_	Domain wall width
*D_SD_*	Critical size of single domain
*D_SP_*	Critical size of superparamagnetic
RMSR	Root-mean-square roughness
OSV	Organic spin valves
OMAR	Organic magnetoresistance
